# Clinical Value of Neutrophil CD64 Index, PCT, and CRP in Acute Pancreatitis Complicated with Abdominal Infection

**DOI:** 10.3390/diagnostics12102409

**Published:** 2022-10-05

**Authors:** Biao Wang, Rongzhu Tang, Shaohong Wu, Ming Liu, Fariha Kanwal, Muhammad Fayyaz ur Rehman, Fang Wu, Jianping Zhu

**Affiliations:** 1 Department of Gastrointestinal Surgery, Renmin Hospital, Hubei University of Medicine, No. 39, Chaoyang Middle Road, Shiyan 442000, China; 2 Department of Gastroenterology, Seventh People’s Hospital of Shanghai University of Traditional Chinese Medicine, No. 358, Datong Road, Pudong New District, Shanghai 200137, China; 3 Department of Critical Care Medicine, Shanghai General Hospital, Shanghai Jiao Tong University School of Medicine, Shanghai 200025, China; 4 Department of Emergency, Shanghai General Hospital, Shanghai Jiao Tong University School of Medicine, Shanghai 200025, China; 5 Med-X Research Institute, School of Biomedical Engineering, Shanghai Jiao Tong University, Shanghai 200240, China; 6 Institute of Chemistry, University of Sargodha, Sargodha 40100, Pakistan; 7 Department of Gynecology, Obstetrics and Gynae Hospital, Fudan University, Shanghai 200437, China

**Keywords:** neutrophil, CD64 index, acute pancreatitis, abdominal infection

## Abstract

Objective: To study the clinical diagnostic value of neutrophil CD64 index, PCT, and CRP in patients with acute pancreatitis with abdominal infection. Methods: A number of patients with acute pancreatitis (*n* = 234) participated in the study. According to the infection and health conditions, they were further divided into the non-infection group (*n* = 122), infection group (*n* = 78), and sepsis group (*n* = 34), and 40 healthy subjects were selected in the control group (*n* = 40). Expression levels of infection indexes, such as CD64 index, PCT, and CRP, were detected and compared. ROC curves were drawn to compare the efficacy of each index in the diagnosis of acute pancreatitis with abdominal infection and sepsis. The study was retrospectively registered under the China Clinical Trial Registry as a trial number ChiCTR2100054308. Results: All indexes were significantly higher in three clinical groups than the healthy control group (*p* < 0.05). The CD64 index, CD64 positive rate, and PCT in the infected group were significantly higher than those in the uninfected group (ALL *p* < 0.05). The PCT of patients infected with Gram-negative bacteria was significantly higher than that of Gram-positive bacteria-infected patients (*p* < 0.05). CD64 index had the best diagnostic efficiency for acute pancreatitis infection, with 82.14% sensitivity, 88.51% specificity, and 0.707 Youden indexes. The CD64 Youden index (0.780) for sepsis diagnosis was the highest, while the AUC of PCT was the highest (0.897). Conclusion: CD64 index combined with PCT has good sensitivity and specificity in diagnosing acute pancreatitis infection and sepsis.

## 1. Introduction

Acute pancreatitis is a common gastrointestinal disease caused by pancreas inflammation due to systemic inflammatory response that may lead to organ or system impairment [1]. It is also characterized by local inflammatory reactions of the pancreas in common clinically acute and severe cases with or without functional changes of other organs. The disease is self-limiting (one week duration) in patients with mild acute pancreatitis [2]. Moderately severe or severe acute pancreatitis (SAP) has the characteristics of rapid progress, dangerous condition, and high clinical mortality [3]. According to revised Atlanta classification (RAC), acute pancreatitis can be either interstitial edematous pancreatitis (IEP) or acute necrotizing pancreatitis (ANP) [4,5,6]. Approximately 20–30% of patients with acute pancreatitis will have necrotizing pancreatitis [7]. According to statistics, the overall mortality of patients with acute pancreatitis is about 5–10%, and that of patients with SAP is 20–30% [8]. While 20% patients of AP may develop SAP, there are two peaks in the course of acute pancreatitis; first stage is systemic inflammatory response syndrome with subsequent organ failure and second is infectious complication stage with related organ failure [9,10]. Without intensive care, SAP may progress into critical acute pancreatitis (CAP) with mortality rate of 34% [11,12,13]. Predicting the risk of complications and carrying out comprehensive treatment in the early stage of acute pancreatitis is the core means to improve the prognosis of patients.

In recent studies, multiple scoring systems have been applied to the severity stratification and early prognosis prediction of patients with acute pancreatitis, but these scores have limitations [14]. Exploring blood markers to predict the condition and complication risk of patients with acute pancreatitis has become an important research direction in recent years. Abdominal infection is an important complication in patients with acute pancreatitis, which can lead to systemic inflammatory response syndrome, multiple organ failure, and septic shock. It is one of the important causes of death in patients with acute pancreatitis [15], but the clinical symptoms and signs of abdominal infection are not specific. However, it is difficult to obtain positive results in the early stage of the disease using imaging diagnosis and etiological examination of peritoneal puncture fluid, thus representing a significant clinical problem.

Although many studies used early infection markers to predict and diagnose abdominal infection in patients with acute pancreatitis, there is no single marker that can meet the clinical needs [16]. It has become a consensus that combining multiple markers can improve the diagnostic effect. The cluster of differentiation 64 (CD64) antigen is considered as an important marker of bacterial infections, systemic inflammation and mortality [17]. It is a transmembrane glycoprotein functioning as a high-affinity IgG receptor (FcγRI) [18]. Cytokines regulate its expression and play a bridge role between humoral immunity and cellular immunity. CD64 is mainly distributed on the surface of monocytes, macrophages, and dendritic cells [19]. Normally, CD64 expression is very low in neutrophils, but the expression is significantly induced in peripheral blood neutrophils during bacterial infection and helps in neutrophil phagocytosis and sterilization [19,20]. Many studies show that CD64 has high specificity in bacterial infection, which is helpful in early diagnosis of bacterial infection and the degree of infection [21,22].

Procalcitonin is another biomarker normally produced in thyroid C cells and enters into blood after its conversion to calcitonin when induced by glucocorticoids, glucagon, gastrin, or β-adrenergic signaling [23]. In infection patients, it is produced by non-thyroid tissues (e.g., adipocytes) upon induction from IL-6 and TNF-α and enter bloods without being converted into calcitonin [24]. Previously, PCT has been reported to be the most sensitive laboratory test for the detection of pancreatitis where low levels of PCT appear to be strong negative predictors of infected necrosis [25]. C-reactive protein (CRP) is a conventional biomarker of systemic inflammatory response (SIRS) and bacterial infections. Based on this current situation, the value of neutrophil CD64 index, PCT, and CRP in predicting the incidence of acute pancreatitis complicated with abdominal infection was analyzed to provide evidence for clinical diagnosis of infectious diseases.

## 2. Materials and Methods

### 2.1. Study Design

**Figure 1:**
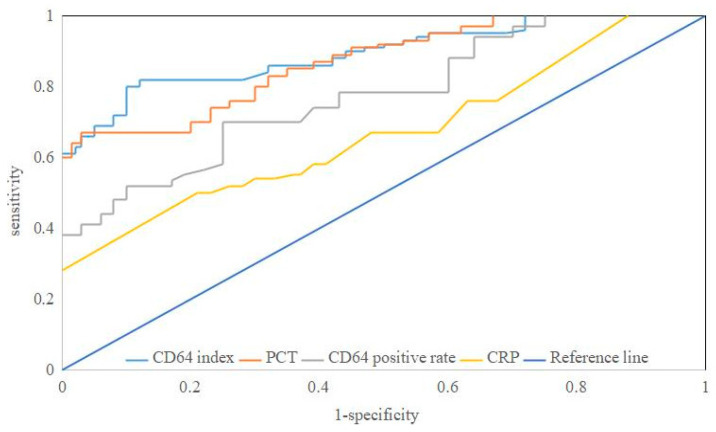
ROC curve of infection indicators for the diagnosis of acute pancreatitis with abdominal infection.

**Scheme 1:**
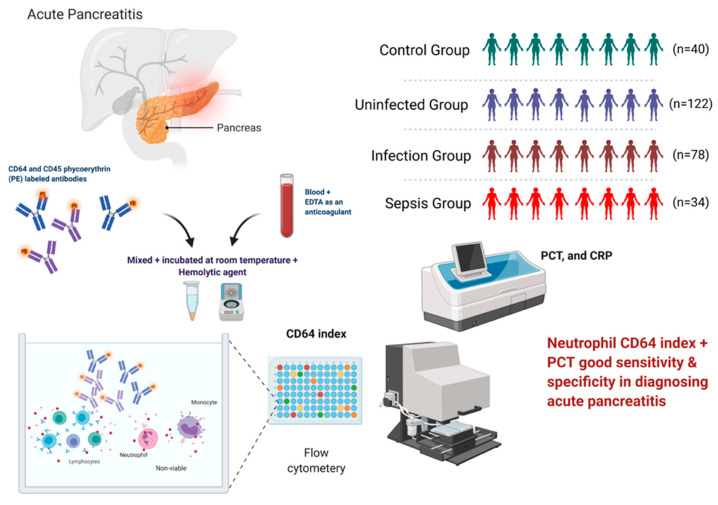
Study design.

A total of two hundred and thirty-four patients with acute pancreatitis and met the inclusion criteria of this study were selected from two class III (class A) hospitals from September 2019 to March 2021. According to the infection, they were divided into the No Infection group (*n* = 122), Infection group (*n* = 78), and Sepsis group (*n* = 34). At the same time, 40 healthy persons who underwent physical examination in our hospital in the same period were selected as the Control group (Figure 1). There was no significant difference between baseline data (all *p* > 0.05), which were comparable (Table 1). All patients signed informed consent and voluntarily participated in the study. The study protocol was approved by the hospital ethics committee (Scheme 1). The study was retrospectively registered under the China Clinical Trial Registry as a trial number ChiCTR2100054308.

#### 2.1.1. Inclusion Criteria

All patients met the diagnostic criteria of acute pancreatitis in the initial treatment guidelines for acute pancreatitis of the American Gastroenterology Association [26], with complete clinical data and age >18 years. Blood samples were obtained from all patients within 3 days of admission. We selected patients strictly according to the inclusion criteria, which can basically ensure the homogeneity of all included patients.

#### 2.1.2. Exclusion Criteria

The study exclusions include patients with malignant tumors, liver and kidney dysfunction, hematological diseases, cardiovascular and cerebrovascular accidents, immune deficiency diseases or autoimmune diseases; patients diagnosed with an infection in other parts or systemic infection except for pancreas and abdominal cavity at admission; patients with diabetes and acute pancreatitis history. There were patients who used glucocorticoids and immunomodulatory drugs three months before enrollment. Pregnant or nursing mother were also excluded.

#### 2.1.3. Diagnostic Criteria for Abdominal Infection

The management guidelines for abdominal infection [27] updated by the World Society of Emergency Surgery (WSES) in 2019 is used as the diagnostic criteria for abdominal infection: patients generally have symptoms and signs, such as abdominal pain, rebound pain, fever, cessation of anal exhaust and defecation, and Balthazar CT grade of acute pancreatitis is grade D or E, and positive blood culture or ultrasound-guided peritoneal puncture fluid pathogen culture.

### 2.2. Flow Cytometric Detection of CD64 Index

The detection and calculation formula of CD64 was analyzed by FACS Calibur flow cytometer (Becton Dickinson, NY, USA), supporting reagents, and software CellQuest (Becton Dickinson, New York, NY, USA). 2 mL of whole blood sample (with EDTA as an anticoagulant) was collected from patients with suspected infection and analyzed within two hours. Samples were mixed upside down eight times and added to the special flow tube before processing. CD64 and CD45 phycoerythrin (PE) labeled antibodies (5 μL each) were added to 50 μL whole blood, mixed well, and incubated at room temperature in the dark for 40 min. A hemolytic agent (500 μL) was added to each tube, mixed well, and incubated for 10 min. The samples were centrifuged for 5 min at 2000× *g*, and the supernatant was discarded. Phosphate buffer (PBS) (mL) was added to the pellet and mixed well before another centrifugation round using the same conditions. After discarding the supernatant, sample pellets were resuspended in 300 μL PBS and analyzed by flow cytometry. CD64 expression on the surface of neutrophils was measured as geometric mean fluorescence intensity (MFI) on neutrophils, namely polymorphonuclear leukocytes (PMN), lymphocytes (LYM), and monocytes (MO). CD64 index was calculated using Leuko64 QuantiCALC Software by the following ratio

CD64 index = (MFI_PMN CD64/MFI_LYM CD64)/(MFI_MO CD64/MFI_PMN CD64)

CD64 positive rate = 100% × Number of CD64 positive PMN cells/total number of PMN cells.

### 2.3. PCT and CRP Index

PCT and CRP were detected from non-anticoagulated peripheral blood by Cobas-e411 Electrochemiluminescence Automatic Immunoanalyzer (Roche, Munich, Germany) and AU5800 Automatic Biochemical Analyzer (Beckman Kurt, Dallas, TX, USA) and their supporting reagents, respectively.

### 2.4. Statistical Analysis

Utilizing SPSS 20.0 software (SPSS Inc., Chicago, IL, USA), the comparison between multiple groups of data adopts one-way ANOVA, the comparison between two groups is subject to Student–Newman–Keuls (SNK) test, and the comparison between two groups of measurement data adopts an independent sample t-test. Comparison row between person data χ^2^ test, Wilcoxon rank-sum test was used to compare grade data. The receiver operator characteristics (ROC) curve was drawn to evaluate the efficacy of various infection indexes in diagnosing acute pancreatitis with abdominal infection and sepsis. *p* < 0.05 was statistically significant. The Youden index [28] measure for the ROC curve was used to measure the overall value of an infection index over the whole region of the ROC curve. Youden index for a diagnostic marker and calculated optimal cut-off point is equivalent to maximizing the sum of sensitivity and specificity for all the possible values of the cut-off point [29].

## 3. Results

### 3.1. Comparison of Infection Indexes

When infection indexes were compared, there was a significant difference among the four groups (all *p* < 0.05). The indexes of patients with acute pancreatitis in the three groups were significantly higher than those in the healthy control group (all *p* < 0.05); The CD64 index, CD64 positive rate, and PCT in the infected group were significantly higher than those in the uninfected group (all *p* < 0.05), but there was no significant difference in CRP between the two groups (*p* > 0.05); All indexes in the sepsis group were significantly higher than those in infection group (all *p* < 0.05), as shown in Table 2.

A comparison of infection indexes among different bacterial infection groups shows that PCT of patients infected with Gram-negative bacteria was significantly higher than that of patients infected with Gram-positive bacterial infections (*p* < 0.05), but there was no significant difference in CD64 index, CD64 positive rate, and CRP indexes between the two groups (all *p* > 0.05), as shown in Table 3.

### 3.2. Evaluation of Diagnostic Efficacy of Acute Pancreatitis with Abdominal Infection

The uninfected acute pancreatitis group is negative, and the infection and sepsis groups of acute pancreatitis are positive. The ROC curve of each index was obtained for diagnosing acute pancreatitis with abdominal infection, as shown in Figure 1. The sensitivity of CD64 index (82.15%), Youden index (0.707), and area under the curve (AUC, 0.892) were greater than other indexes, while the diagnostic specificity of PCT (95.11%) was the highest (Table 4).

### 3.3. Evaluation of Diagnostic Efficacy of Acute Pancreatitis with Sepsis

**Figure 2:**
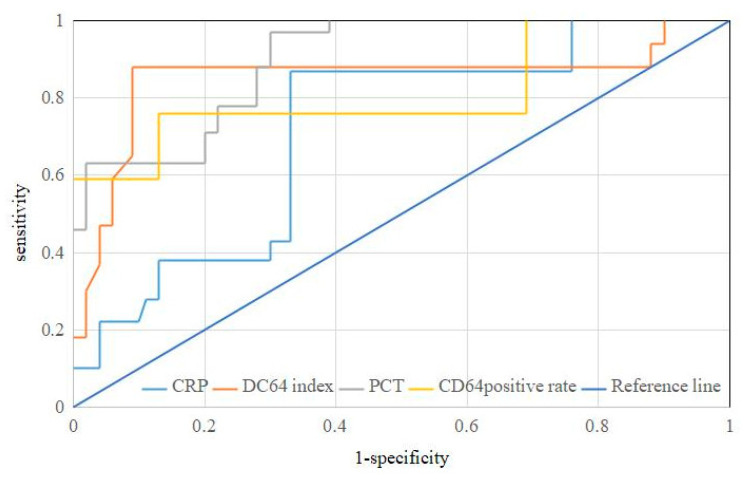
ROC curves of infection indicators for the diagnosis of acute pancreatitis and sepsis.

In this case, the acute pancreatitis infection group is negative, and the sepsis group is positive. The ROC curve of each index for the diagnosis of burn sepsis is drawn (Figure 2). Among them, the CD64 index and Youden index (0.780) are the largest, while the AUC of PCT (0.897) is the highest (Table 5).

### 3.4. Combined Diagnosis

The above CD64 index and PCT with good diagnostic effects are used for combined diagnosis, and the positive value is greater than the optimal critical diagnostic value. The sensitivity and Youden index are higher than the single index (Table 6).

## 4. Discussion

Acute pancreatitis is one of the common emergencies of the digestive system, including mild-acute and severe-acute pancreatitis. Later is prone to septic shock and organ dysfunction. Symptoms and signs or imaging examinations are ineffective in diagnosing acute pancreatitis complicated with abdominal infection. In the clinic, a large number of patients often delay treatment and cause a bad prognosis. So, the mortality of secondary infection is as high as 36–50%. Some patients with acute pancreatitis can suffer from sequelae of pancreatitis [30,31,32].

Bacteriological culture results and drug sensitivity tests are the gold standards for diagnosing bacterial infection and guiding antibiotic treatment. Still, their positive rate is low, and the isolation time of pathogens is long, which cannot provide the basis for early diagnosis in time. Therefore, finding a rapid and accurate method to evaluate a bacterial infection is particularly important.

In recent years, the application of flow cytometry to detect the expression of CD64 on the surface of peripheral blood neutrophils has attracted extensive attention in the diagnosis of bacterial infection. CD64 mRNA in neutrophils began to express 1~3 h after infection, and CD64 on the cell surface could be detected up-regulated 3~6 h after infection, which has the ability to detect infection early [33]. Rogina et al. also showed that the differentiation of CD64 index and CD64 positive rate in each group of acute pancreatitis was higher. The CD64 index, CD64 positive rate, and PCT in the infected group were significantly higher than those in the uninfected group (all *p* < 0.05), but there was no significant difference in CRP between the two groups (*p* > 0.05). It may be that CRP primarily reflects the acute stress state in vivo, and the basic CRP in patients with acute pancreatitis is higher. The increase was not obvious during infection. Previously, CRP shows low specificity and limited correlation with the disease activity in comparison to other infection indexes [34].

Some studies pointed out that Gram-negative bacteria infection in patients with acute pancreatitis with abdominal infection was significantly higher than that of Gram-positive bacteria [35]. Therefore, this study compared the differences of various indexes between the two types of bacterial infections. The results showed that the PCT of Gram-negative bacterial infections was significantly higher than that of Gram-positive bacterial infections (*p* < 0.05), but there was no significant difference in CD64 index, CD64 positive rate, and CRP between the two groups (all *p* > 0.05). PCT is an important biomarker commonly used to predict bacterial infections in clinics. It can also guide the diagnosis and treatment of infectious diseases. Its sensitivity and specificity are higher than traditional markers such as WBC and CRP. CRP test is controversial in term of limitation sensitivity and specificity [36]. PCT may be better than CD64 index in the early diagnosis of bacterial infection [37] but CD64 index is still preferable and a method of choice in diagnosing severe and complicated bacterial infection in term of sensitivity and specificity [38,39]. When the body is infected or invaded by endotoxins, neutrophil CD64 can increase 4~6 h after stimulation and 0~24 h after sepsis. This study confirmed by the bacterial culture that the peripheral blood CD64 index of acute pancreatitis (AP) patients can be significantly increased within 24 h after concurrent bacterial infection, which is expected to become a new biomarker for the early diagnosis of sepsis.

Ye Z et al. reported that the sensitivity of CD64 on the surface of neutrophils for the diagnosis of bacterial infection is ≥90%, and the specificity can reach 90~100%, which is significantly better than PCT, CRP, and other indicators. However, there are many ways to express the test results, including CD64 MFI, CD64 positive rate, and CD64 index, and the calculation formula of CD64 index is not unified. The calculation formula of the CD64 index for diagnosing infection obtained by various laboratories is quite different from the cut-off value, and the comparability is poor [40].

Because CD64 is expressed in various cells; in monocytes, these are produced in large amounts in physiological and infectious states, while lymphocytes have low expression of CD64 [41]. Considering this expression pattern, this study used (MFI_PMN CD64/MFI_LYM CD64)/(MFI_MO CD64/MFI_PMN CD64) to express the CD64 index, which not only quantified the expression level of CD64, but could also reduce the operation error to make the CD64 index more objective. The results showed that the efficacy of CD64 index in the diagnosis of acute pancreatitis with abdominal infection was better than other indexes, but the specificity was lower than PCT. The Youden index (0.781) was the largest in the diagnosis of sepsis, while the AUC of PCT (0.897) was the highest. The sensitivity of the CD64 index was higher than that of PCT, but the specificity was lower than that of PCT. Previously, the sensitivity and specificity of neutrophil CD64 was found to be more than 80% [42].

When the CD64 index was combined with PCT, the sensitivity and Youden index increased, but the specificity decreased. It is suggested that combined diagnosis is helpful to improve the diagnostic efficiency of acute pancreatitis with abdominal infection and sepsis.

## 5. Conclusions

In conclusion, neutrophil CD64 index combined with PCT has good sensitivity and specificity in diagnosing acute pancreatitis with abdominal infection and sepsis and has a good prospect of clinical application.

## Figures and Tables

**Table 1:** Comparison of basic data of patients in each group.

Group	*n*	Sex [*n* (%)]		Age (Years)	Etiology [*n* (%)]		
		Male	Female	(x ± s)	Biliary	Hyperlipidemia	Other Reasons
Control group	40	27 (67.50)	13 (32.50)	45.21 ± 8.24			
Uninfected group	122	74 (60.66)	48 (39.34)	44.49 ± 9.39	66 (54.10)	38 (31.15)	18 (14.75)
Infection group	78	46 (58.97)	32 (41.03)	43.61 ± 9.47	50 (64.10)	20 (25.64)	8 (10.26)
Sepsis group	34	22 (64.71)	12 (35.29)	44.21 ± 8.26	22 (64.71)	6 (17.65)	6 (17.65)
Statistical test		X^2^ = 1.001		F = 0.592	X^2^ = 4.089		
*p*-value		0.801		0.555	0.394		

**Table 2:** Comparison of infection indexes in 4 groups.

Group	*n*	CD64 Positive Rate	CD64 Index	CRP (mg/L)	PCT (μg/L)
Control group	40	9.79± 3.56	0.76± 0.21	2.29 ± 0.81	0.12± 0.07
Uninfected group	122	23.41 ± 7.17 ^a^	1.21 ± 0.35 ^a^	24.43± 4.61 ^a^	1.14 ± 0.78 ^a^
Infection group	78	43.91 ±15.82 ^ab^	3.34 ± 0.94 ^ab^	25.51 ±4.97 ^a^	2.53 ± 1.25 ^ab^
Sepsis group	34	67.71 ±9.64 ^abc^	5.06 ± 0.36 ^abc^	29.74 ±4.64 ^abc^	5.35 ± 1.75 ^abc^
F-value		177.321	304.560	328.296	131.643
*p*-value		<0.001	<0.001	<0.001	<0.001

Note: Compared with the control group, ^a^ *p* < 0.05; Compared with the uninfected group, ^b^ *p* < 0.05; Compared with the infected group, ^c^ *p* < 0.05.

**Table 3:** Comparison of infection indexes between different bacterial infection groups.

Bacteria Types	*n*	CD64 Ositive Rate	CD64 Index	CRP (mg/L)	PCT (μg/L)
Gram-negative bacteria	89	51.91 ± 12.78	4.02 ± 0.81	27.36 ± 5.19	3.91 ±1.11
Gram-positive bacteria	23	49.93 ± 10.55	3.75 ± 0.76	25.954 ± 4.74	2.64 ± 0.75
F-value		0.621	1.259	1.031	4.802
*p*-value		0.550	0.239	0.329	<0.001

**Table 4:** Diagnostic efficacy of infection indicators for acute pancreatitis complicated with abdominal infection.

Infection Index	AUC *	Sensitivity (%)	Specificity (%)	Youden Index
CD64 positive rate	0.669	71.44	75.42	0.469
CD64 index	0.892	82.15	88.51	0.707
CRP	0.622	58.94	81.96	0.409
PCT	0.867	67.85	95.11	0.630

* AUC; area under the curve.

**Table 5:** Diagnostic efficacy of infection indicators for acute pancreatitis complicated with sepsis.

Infection Index	AUC	Sensitivity (%)	Specificity (%)	Youden Index
CD64 positive rate	0.815	76.48	87.19	0.637
CD64 index	0.847	88.26	89.76	0.780
CRP	0.627	88.25	66.69	0.549
PCT	0.897	70.61	94.87	0.655

**Table 6:** CD64 index and PCT combined diagnostic efficiency.

Diagnosis of Content	Sensitivity (%)	Specificity (%)	Youden Index
Acute pancreatitis with abdominal infection	89.29 (100/112)	88.52 (108/122)	0.778
Acute pancreatitis with sepsis	94.12 (32/34)	84.62 (66/78)	0.787
